# Gallic acid suppresses esophageal squamous cell carcinoma progression and enhances cisplatin chemosensitivity through IL-6/STAT3/Notch pathway

**DOI:** 10.32604/or.2025.060151

**Published:** 2025-05-29

**Authors:** NURAN BEDOLLA, HAO WU, LINYU LIU, XUETING LIU, YANLI REN

**Affiliations:** College of Biological Sciences and Technology, YiLi Normal University, Yining, 835000, China

**Keywords:** Gallic acid (GA), Cisplatin, Esophageal cancer, Interleukin-6 (IL-6), Chemotherapy sensitivity, Signal transducer and activator of transcription 3 (STAT3)/Notch pathway

## Abstract

**Background:**

Gallic acid (GA), a plant-derived polyphenol, possesses diverse biological functions such as reducing inflammation and against tumors. Currently, the influence of GA on the resistance of esophageal squamous cell carcinoma (ESCC) cells to cisplatin (DDP) is not well understood.

**Methods:**

Cell counting kit-8 assay examined how GA affected KYSE30 and TE-1 cell viability. 5-Ethynyl-2′-deoxyuridine and TdT-mediated dUTP Nick-End labeling staining detected cell proliferation and apoptosis. Clone formation assay, flow cytometry, Carboxyfluorescein diacetate succinimidyl ester fluorescent probes, and Transwell assay determined cell biological properties, and 2′,7′-Dichlorofluorescin diacetate (DCFH-DA) fluorescent probes detected oxidative stress levels. Signal transducer and activator of transcription 3 (STAT3)/Notch pathway protein levels after GA and/or Interleukin-6 (IL-6) intervention were examined through Western blot. Furthermore, a model for subcutaneous graft tumors was established in nude mice.

**Results:**

GA exerted suppressive effects on cell proliferation, and caused apoptosis of KYSE30 and TE-1 cells. IL-6 intervention activated the STAT3/Notch pathway and promoted the malignant biological properties of ESCC cells. In contrast, GA attenuated the effects of IL-6, while STAT3 or Notch inhibitor further enhanced the effects of GA, suggesting that GA inhibited the IL-6/STAT3/Notch pathway. Not only that, GA promoted oxidative stress and enhanced cell sensitivity to DDP both *in vitro* and *in vivo*.

**Conclusion:**

GA suppresses the malignant progression of ESCC and enhances cell sensitivity to DDP by hindering the IL-6/STAT3/Notch pathway.

## Abbreviations

DDP: Cisplatin
ESCC: Esophageal squamous cell carcinoma
EC: Esophageal carcinoma
EAC: Esophageal adenocarcinoma
GA: Gallic acid
IL-6: Interleukin-6
STAT3: Signal transducer and activator of transcription 3
ROS: Reactive oxygen species
CCK-8: Cell Counting Kit-8
PI: Propidium iodide
IL-6: Interleukin-6
EdU: 5-Ethynyl-2′-deoxyuridine
TUNEL: TdT-mediated dUTP Nick-End labeling
DCFH-DA: 2′,7′-Dichlorofluorescin diacetate
CFSE: Carboxyfluorescein diacetate succinimidyl ester
BSA: Bovine serum albumin
MMP: Matrix metalloproteinase
HES1: Hairy and enhancer of split 1
HEEC: Human normal esophageal epithelial cells

**Highlights:**

1. Gallic acid (GA) inhibits proliferation and induces apoptosis of ESCC cells.

2. Interleukin-6 (IL-6) intervention activates the Signal transducer and activator of transcription 3 (STAT3)/Notch pathway and it promotes ESCC malignant progression and increases cell resistance to cisplatin (DDP).

3. GA modulates the IL-6/STAT3/Notch pathway, inhibits ESCC malignant progression, and promotes oxidative stress.

4. The combination of GA and DDP shows increased sensitivity of ESCC cells to DDP.

5. The IL-6/STAT3/Notch pathway is suppressed by GA, leading to increased sensitivity of ESCC cells to DDP.

## Introduction

Globally, esophageal carcinoma (EC) is a prevalent form of cancer, ranking sixth in terms of total deaths compared to other cancer types [1]. EC prevalence in China is the highest in the world [2]. In 2020, China reported 324,000 and 301,000 new EC cases and deaths, respectively, with a global share of 53.70% and 55.35% [3]. Esophageal squamous cell carcinoma (ESCC) is the most prevalent type of pathology, representing around 90% of all EC cases [4,5]. Because of the lack of specificity in the clinical symptoms and indicators of early ESCC, many patients are first diagnosed when the disease has progressed in the mid-advanced stage, leading to a bleak prognosis, with a 5-year survival rate falling below 20% [6,7]. Consequently, discovering new and efficient therapeutic strategies is particularly important to improve the outcomes of ESCC patients.

Chemotherapy is one of the mainstays in the management of ESCC patients in the mid-advanced stage, of which platinum-based drugs have been commonly utilized in ESCC treatment [8–10]. Since its approval for use in the late 1970s, cisplatin (DDP) has become the main chemotherapeutic agent for the management of various types of tumors [11,12]. The platinum atoms of DDP cross-link with DNA to form adducts that cause damage to DNA and proteins in cancer cells and antagonize DNA replication and transcription [13]. In addition, DDP could generate elevated reactive oxygen species (ROS) levels, causing oxidative stress in cancer cells [14]. Although DDP chemotherapy is an important therapeutic option for ESCC, most patients eventually relapse or develop resistance, and long-term outcomes are unsatisfactory [15,16]. Therefore, the current research is centered on enhancing ESCC sensitivity to DDP chemotherapy to enhance treatment outcomes.

Gallic acid (GA), with a molecular formula of C_7_H_6_O_5_, is a natural plant polyphenol compound, that has various physiological effects like antioxidant, antibacterial and anticancer activities [17,18]. Sun et al. reported that GA-suppressed proliferation promoted apoptosis of ESCC cells, and effectively hindered tumor growth in mice [19]. Notably, GA reduced the cell viability, and when combined with DDP, it showed a notably greater inhibitory impact on ovarian cancer, suggesting that it could increase the DDP sensitivity of ovarian cancer cells [20]. However, it is unclear whether GA improves the DDP sensitivity of ESCC.

Interleukin-6 (IL-6) is essential for regulating autoimmune responses and is linked to the advancement of tumors [21]. High levels of IL-6 cause a Signal transducer and activator of transcription 3 (STAT3) phosphorylation, which activates the Notch signaling pathway [22]. Notch signaling regulates immune cell development and function, maintains rapid proliferation of cancer cells, and has a significant impact on cancer advancement [23]. Furthermore, suppression of STAT3 phosphorylation blocked the link between IL-6 and Notch signaling pathway [24]. As a result, we hypothesized that GA improves the DDP sensitivity of ESCC cells by modulating the IL-6/STAT3/Notch pathway. Based on this hypothesis, this study explored the impacts of GA, IL-6, or DDP on cell malignant biological properties. In addition, a nude mice subcutaneous transplantation tumor model was constructed to clarify the influence of GA *in vivo*. The purpose of this research was to explore the role of GA in the regulation of DDP resistance in ESCC cells, with a view to offer a new reference for the clinical application of GA and ESCC management.

## Materials and Methods

### Cell culture and processing

Human normal esophageal epithelial cells (HEEC, CP-H031), ESCC cell lines KYSE30 (CL-0577) and TE-1 (CL-0577) were obtained from Priscilla Biotechnology Co., Ltd. (Wuhan, China), and were free of mycoplasma contamination. Before the experiments, cells were maintained in RPMI-1640 medium (11875119, Gibco, Grand Island, NY, USA), 1% penicillin/streptomycin double antibiotics (15140122, Gibco, Grand Island, NY, USA), with 10% fetal bovine serum (A5670701, Gibco, Grand Island, NY, USA). The interval between fluid changes was 3 days, and the cultivation environment was at 37°C with 5% CO_2_ by volume contained.

In the GA (HY-N0523, MedChemExpress, Monmouth Junction, NJ, USA) cytotoxicity assay, cells were exposed to 25, 50, 100, 200, 300, 400, or 500 μM of GA for 24 or 48 h. In the subsequent ESCC cell biological assay, the IL-6 group medium contained IL-6 (50 ng/mL, I9646, Sigma-Aldrich, St. Louis, MO, USA) and the IL-6+GA group medium contained IL-6 (50 ng/mL) + GA (100 μM). The IL-6+GA+S31-201 group was based on the IL-6+GA group with the addition of the 10 μM of STAT3 inhibitor S31-201 (HY-15146, MedChemExpress, Monmouth Junction, NJ, USA), while the IL-6+GA+DAPT group was based on the IL-6+GA group with the addition of the Notch inhibitor DAPT (20 μM, HY 13027, MedChemExpress, Monmouth Junction, NJ, USA), and incubation time was 24 h in all groups.

### Cell Counting Kit-8 (CCK-8) assay

ESCC cells were inoculated into 96-well cell culture plates (1.5 × 10^4^ cells/well) when the cells were had fully adhered, incubated for 24 or 48 h, and 10% CCK-8 reagent (C0038, Beyotime, Shanghai, China) was introduced into every well. After a 2-h incubation period at 37°C, OD_450_ values were determined with an enzyme marker (1410101, Thermo Fisher Scientific, Waltham, MA, USA). In addition, ESCC cells after different treatments were added to a medium containing DDP (2.5, 5, 10, or 20 μM, HY-17394, MedChemExpress, Monmouth Junction, NJ, USA). Following a 48-h incubation, the OD_450_ values of these cells were determined according to the above procedure, and the IC50 of the cells to DDP was computed.

### 5-Ethynyl-2′-deoxyuridine (EdU) staining

EdU cell proliferation detection kit (C0071S, Beyotime, Shanghai, China) was utilized to evaluate the proliferation of ESCC cells. Cells from different treatment groups were incubated with EdU culture solution (10 μM) in the dark for 60 min. Following two rinses with PBS, cells were exposed to 4% paraformaldehyde (P1110, Solarbio, Beijing, China) for 15 min. Added PBS containing 0.3% Triton X-100 (X100, Sigma-Aldrich, St. Louis, MO, USA) to permeabilize for 10 min, and incubated with Click solution in the dark for 30 min. After that, incubated with DAPI staining solution (D9542, Sigma-Aldrich, St. Louis, MO, USA) for 10 min, and blocked with anti-fade mounting medium (HY-K1042, MedChemExpress, Monmouth Junction, NJ, USA). The observation and photography were conducted using an inverted fluorescence microscope (EVOS, Thermo Fisher Scientific, Waltham, MA, USA).

### TdT-mediated dUTP Nick-End labeling (TUNEL) staining

Cells from different treatment groups were exposed to 4% paraformaldehyde for half an hour, then incubated with 0.3% Triton X-100 for 5 min. Following two rinses using PBS, TUNEL assay solution (50 μL, C1086, Beyotime, Shanghai, China) was gently added dropwise to evenly cover the cells and incubated for 90 min in the dark. After that, the product was incubated with DAPI solution for 10 min without light. After anti-fade blocking, a fluorescence microscope was employed for observation and imaging.

### Clone formation assay

Cells were PBS-washed and digested with 0.25% trypsin (HY-K3009, MedChemExpress, Monmouth Junction, NJ, USA) to obtain individual cells. 500 cells to be added into each well of the 6-well plate with culture medium corresponding to different treatment groups, and cultured (37°C, 5% CO_2_) for 14 day. The culture medium was refreshed with a new one every two to three days. When clonal cell clusters were observable to the naked eye, the culture was stopped. The culture solution was aspirated and rinsed twice with PBS. Cells were exposed to 4% paraformaldehyde and fixed for 20 min. The fixative was discarded, and exposed to 0.1% crystal violet (C0121, Beyotime, Shanghai, China) for 10 min. Photographs were taken with a fluorescent inverted microscope and then calculated the clone formation rate.

### Flow cytometry

After various treatments, cells were gathered and gently mixed with Binding Buffer (500 μL). Following that, incubated with propidium iodide (PI, 5 μL, ST1569, Beyotime, Shanghai, China) and Annexin-V-FITC (5 μL, HY-K1073, MedChemExpress, Monmouth Junction, NJ, USA) in darkness for 15 min. Cell apoptosis was identified by flow cytometry (BD FACSCaliburTM, BD biosciences, San Jose, CA, USA).

### Cell cycle assay

Cells were seeded into a 6-well plate (1 × 10^6^ cells/well). Following cell attachment, different treatment groups of medium were introduced. After 24 h, cells were gathered and mixed well with 1 mL of cell fixation solution (70% ethanol) and incubated at 4°C for 12 h. To prepare the cell staining solution, RNaseA (200 μg/mL, ST579, Beyotime, Shanghai, China) and PI (50 μg/mL) were mixed in PBS. Added cell staining solution (500 μL) to thoroughly resuspend the cells, and incubated in the dark for half an hour. A flow cytometer was utilized to examine fluorescence, while the analysis of the cell cycle was performed with FlowJo software (v10.8, BD Biosciences, San Jose, CA, USA).

### Carboxyfluorescein diacetate succinimidyl ester (CFSE) staining

ESCC cells from different treatments were diluted in PBS, added CFSE solution (5 μM, C1031, Beyotime, Shanghai, China) into cell suspension (1 × 10^6^ cells/mL), and stained for 10 min at 37°C. Added 5 times the volume of the complete medium, mixed well, and terminated the staining. Centrifuged and discarded the supernatant, subsequently rinsed with PBS twice to remove unbound CFSE solution. Resuspended with PBS buffer, a small amount of drops and added to the 96-well plate, and observed under the fluorescence microscope to see if the labeling was successful. The CFSE-pre-stained cells were taken and added to the culture medium, and cell proliferation was examined using flow cytometry after 24 h of incubation.

### Transwell

The Matrigel matrix gel (354230, Corning, Tewksbury, MA, USA) was placed in the freezer layer of the refrigerator for melting and removal. The matrix gel was diluted in serum-free medium, which was then added (100 μL) to each Transwell (Corning, Tewksbury, MA, USA) and subsequently incubated overnight. The upper chamber was filled with 200 μL of cell suspension (1.5 × 10^5^ cells/mL), and subsequently, the complete culture medium for various treatment groups was added to the lower chamber. After incubation for 24 h, the cells that had not passed through the membrane were removed by cotton swabs. Subsequently, exposed to 4% paraformaldehyde for 30 min, and dyed with 0.1% crystal violet. A random field was chosen and observed using an inverted fluorescence microscope to quantify invasive cell numbers.

The Transwell migration test does not require Matrigel matrix gel, and the rest of the manipulation and analytical steps are consistent with the invasion test.

### ROS test

KYSE30 and TE-1 cells were grown on sterile coverslips and exposed to different media for 24 h. The coverslips were removed and the excess media was aspirated away. Added 100 μL 2′,7′-Dichlorofluorescin diacetate (DCFH-DA) fluorescent probe (10 μM, HY-D0940, MedChemExpress, Monmouth Junction, NJ, USA) to uniformly cover the cell coverslips and allowed to incubate for 20 min in darkness. Afterward, PBS was used to rinse the sample twice, observed using a fluorescence microscope. Image J software (version 1.54 h, Wavne Resband, National Institute of Mental Health, USA) processed the images and assessed ROS levels.

### Establishment of drug-resistant cell line TE-1/DDP

TE-1 cells were exposed to a culture medium containing a two-fold IC50 concentration of DDP that was two times higher and then cultured for 2 to 3 days. Only a small number of cells survived, and apoptotic cells and cell debris were washed away. The original medium was exchanged for medium containing IC50 concentration of DDP, and the interval between fluid changes was 2~3 days. After the cells had grown to cover about 80% of the area at the bottom of the Petri dish, the medium was exchanged for a four-fold IC50 concentration of DDP, and cultured for 2~3 days. Apoptotic cells and their fragments were washed away. The medium was again exchanged for medium containing IC50 concentration of DDP. Repeated the above steps for about 3 months to obtain a drug-resistant cell (TE-1/DDP) that is able to maintain a good growth state in a medium containing IC50 concentration of DDP and can continue to propagate after passaging [25,26].

### Establishment of subcutaneous tumor model

BALB/c nude mice (12–15 g), male or female, aged 4~6 weeks, were obtained from Vitalriver (Beijing, China) and placed in an SPF-grade 22°C constant temperature environment. 0.2 mL of TE-1/DDP cell suspension (1 × 10^7^ cells/mice) was injected subcutaneously in the right axilla of the nude mouse to establish a subcutaneous tumor model. Once the tumors reached 50–100 mm^3^, mice were separated randomly into model groups (Blank), DDP group, DDP+GA group, IL-6 group, IL-6+DDP group, IL-6+GA group, and each group had 6 mice. Each group of mice was intraperitoneally injected with 0.3 mL of saline, DDP (4 mg/kg), DDP (4 mg/kg) + GA (80 mg/kg), IL-6 (5 μg/kg), IL-6 (5 μg/kg) + DDP (4 mg/kg), IL-6 (5 μg/kg) + GA (80 mg/kg), and was administered once every 3 days, respectively. The subcutaneous tumor size in nude mice was evaluated on days 7, 14, and 21 using vernier calipers and the volume of the tumors was determined, volume = π/6 × long diameter × short diameter × height [27]. After 21 days of drug treatment, mice were anesthetized and euthanized, with their tumors being surgically removed and photographed for records. This study received approval from the YiLi Normal University Experimental Animal Ethics Committee (No. ZK-20210608).

### Immunohistochemistry

Tumor tissues were exposed to 4% paraformaldehyde to fix, then routinely dehydrated, and sliced after being embedded in paraffin (4~5 μM thick), deparaffinized using xylene (247642, Sigma-Aldrich, St. Louis, MO, USA). After being rinsed three times with PBS and slightly dried, the slices had a circle drawn around them using a histochemical pen. A drop of 0.3% Triton X-100 was added to cover the tissue, and the slices were incubated for 20 min. Added 3% H_2_O_2_ solution to cover the tissue for 10 min [28]. Following this, the tissues were thoroughly covered with 5% bovine serum albumin (BSA, V900933, Sigma-Aldrich, St. Louis, MO, USA), and closed for half an hour. Added Cleaved caspase-3 primary antibody (ab32024, 1:200, Abcam, Cambridge, MA, USA) or Ki67 primary antibody (ab15580, 1:1000, Abcam, Cambridge, MA, USA) to the sections and incubated for 1.5 h at 37°C. Following that, incubated with HRP-labeled goat anti-rabbit IgG (31460, 1:10,000, Invitrogen, Carlsbad, CA, USA) for 20 min at 37°C. Using DAB solution (DA1010, Solarbio, Beijing, China) to develop color, and using tap water to stop the reaction. Re-stained using Mayer Hematoxylin solution (MHS16, Sigma-Aldrich, St. Louis, MO, USA), sealed with neutral gum, and observed by microscope.

### Western blot

To extract proteins from cells or tissues, RIPA lysis buffer (P0013B, Beyotime, Shanghai, China) was applied, and a BCA kit (P0012, Beyotime, Shanghai, China) was for determine protein contents. The samples underwent electrophoresis on SDS-PAGE gels (12%, WBT41220BOX, Invitrogen, Carlsbad, CA, USA), shifted to a polyvinylidene fluoride membrane (88585, Invitrogen, Carlsbad, CA, USA), and blocked with 5% BSA for 3 h. Following the washing of the membranes, they were treated with primary antibodies, like p-STAT3 (44-384G, 1:1000, Invitrogen, Carlsbad, CA, USA), STAT3 (ab68153, 1:2000, Abcam, Cambridge, MA, USA), hairy and enhancer of split 1 (HES1, ab108937, 1:1000, Abcam, Cambridge, MA, USA), Notch1 (ab52627, 1:2000, Abcam, Cambridge, MA, USA), Cleaved-caspase 3 (PA5-30622, 1:3000, Invitrogen, Carlsbad, CA, USA), matrix metalloproteinase (MMP)-2 (ab97779, 1:500, Abcam, Cambridge, MA, USA), Bcl-2 (ab59348, 1:500, Abcam, Cambridge, MA, USA), MMP-9 (ab76003, 1:300, Abcam, Cambridge, MA, USA), and Bax (ab53154, 1:100, Abcam, Cambridge, MA, USA) at 4°C for overnight. On the next day, the membranes were washed thrice and then cultured with goat anti-rabbit IgG (1:10,000) for 2 h. Added ECL chemiluminescent agent (34577, Invitrogen, Carlsbad, CA, USA) to the membrane and visualized it using a gel imaging system (iBright CL1500, Thermo Fisher Scientific, Waltham, MA, USA). The grayscale values were quantified through Image J software, and the relative protein level was assessed by comparing it to GAPDH (MA5-15738, 1:1000, Invitrogen, Carlsbad, CA, USA).

### Statistical analysis

Each experiment was conducted at least three times, and the findings were presented as mean ± standard deviation. SPSS 26.0 software (IBM, Armonk, NY, USA) to analyze the data statistically, Student’s *t*-test evaluated the distinctions between the two groups. One-way analysis of variance (ANOVA) was conducted to compare multiple groups. Prism software (GraphPad 9.0, GRAPHPAD SOFTWARE, LLC, San Diego, CA, USA) was utilized for plotting images. *p < 0.05 denotes a significant difference.

## Results

### GA hinders ESCC cell proliferation

**Figure 1:**
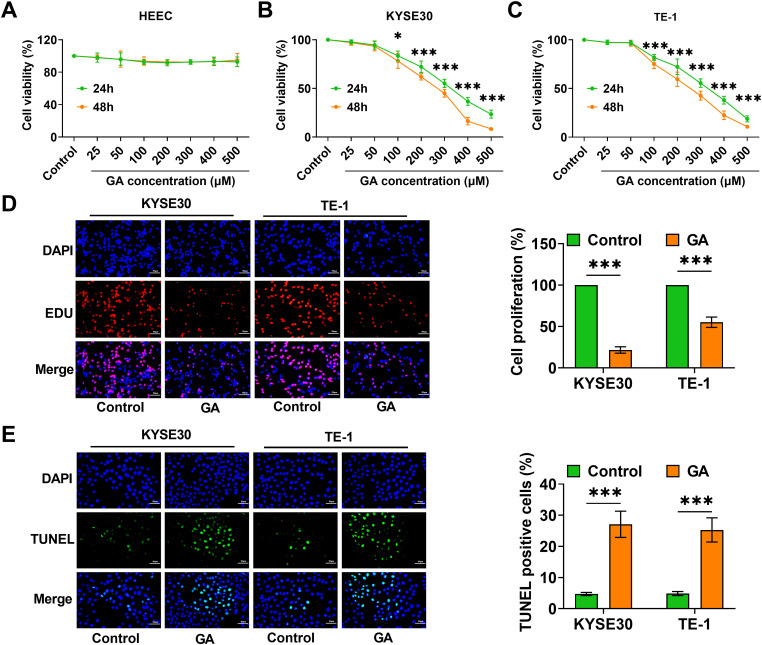
GA inhibits ESCC cell growth. (A–C) CCK-8 assay evaluated cell viability after treatments with GA to identify the optimal concentration and optimal treatment time. (D) EdU staining detected cell proliferation after being exposed to 100 μM GA for 24 h (40×, bar = 50 μM). (E) TUNEL staining evaluated the cell apoptosis (40×, bar = 50 μM) (*p < 0.05, ***p < 0.001).

The impact of varying concentrations of GA on the cell viability of HEEC, TE-1, and KYSE30 cells was evaluated using CCK-8 assay. Treatment with GA at 100, 200, 300, 400, or 500 μM showed no significant reduction in HEEC cell viability, while it notably declined the viability of KYSE30 and TE-1 cells, and the effect was concentration- and time-dependent (Fig. 1A–C). Since 100 μM of GA treatment for 24 h resulted in noticeable inhibition, we decided to use the same treatment condition for the following experiments. Following exposure to GA (100 μM) for 24 h, EdU staining findings showed a notable decline in EdU-positive cell numbers, indicating the suppressive impact of GA on cell proliferation (Fig. 1D). In addition, GA treatment caused a notable rise in TUNEL-positive cell numbers in KYSE30 and TE-1 cells, indicating that GA induces cell apoptosis (Fig. 1E). The findings validated that GA hindered the growth of ESCC cells and stimulated cell apoptosis.

### GA could modulate the IL-6/STAT3/Notch pathway

**Figure 2:**
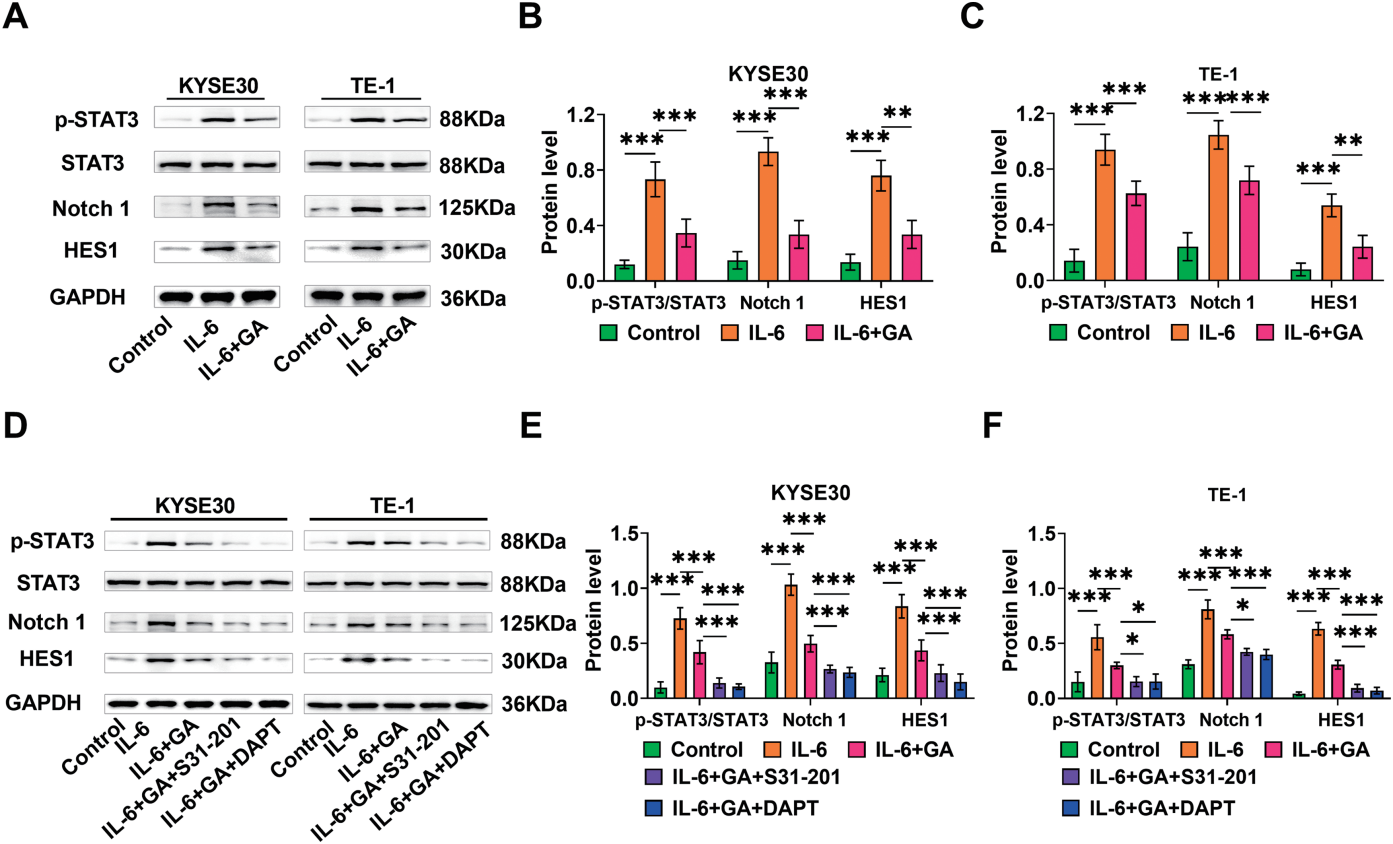
GA modulates the IL-6/STAT3/Notch pathway. (A–C) Examining the levels of pathway proteins p-STAT3/STAT3, Notch 1, and HES1 in KYSE30 and TE-1 cells through Western blot. (D–F) Western blot detected p-STAT3/STAT3, Notch 1, and HES1 protein levels after the addition of Notch inhibitor DAPT or STAT3 inhibitor S31-201 (*p < 0.05, **p < 0.01, ***p < 0.001).

To explore how GA suppressed ESCC cell proliferation, we evaluated the relevant pathways. ESCC cells were exposed to GA and 50 ng/mL of IL-6, and STAT3/Notch pathway protein levels were assessed by Western blot. After a 24-h exposure to IL-6, the levels of Notch1 and HES1 proteins as well as STAT3 phosphorylation were notably increased in KYSE30 and TE-1 cells, indicating that IL-6 activated the STAT3/Notch pathway. The levels of Notch1 and HES1 proteins as well as STAT3 phosphorylation were reduced by the addition of GA, and the effect of IL-6 could be weakened at the same time, suggesting that GA could regulate the IL-6/STAT3/Notch pathway (Fig. 2A–C). Next, to confirm the regulatory impact of GA on the IL-6/STAT3/Notch pathway, we added the STAT3 inhibitor S31-201 or the Notch inhibitor DAPT to the culture solution of GA+IL-6. We found that the levels of Notch1 and HES1 proteins as well as STAT3 phosphorylation further reduced in KYSE30 and TE-1 cells upon the addition of S31-201 or DAPT, which further confirmed that GA regulates IL-6/STAT3/Notch pathway (Fig. 2D–F).

### GA inhibits ESCC cell proliferation, invasion, and migration by IL-6/STAT3/Notch pathway

**Figure 3:**
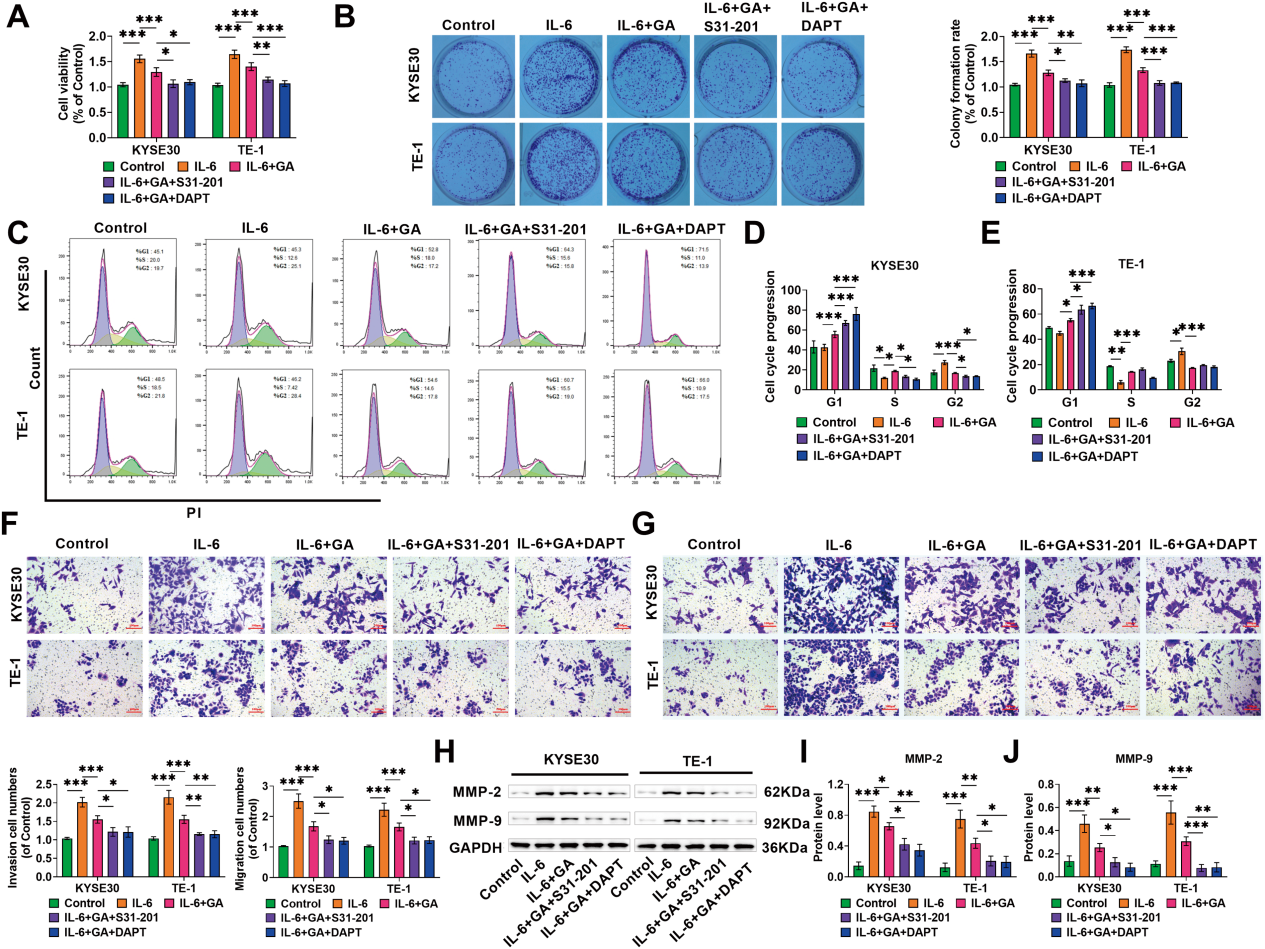
GA suppresses ESCC cell proliferation, invasion, and migration by the IL-6/STAT3/Notch pathway. (A) After different treatments, cell viability was examined using CCK-8 assay. (B) Clone formation assay evaluated that GA suppressed cell proliferation. (C–E) Flow cytometry measured that GA caused a rise in G1 phase and S phase cell numbers, and a decline in the G2 phase, S31-201 or DAPT further caused cells to arrest in the G1 phase. (F–G) Transwell assay examined invaded and migrated cells, with the amount of invasive and migrated cells counted (20×, bar = 100 μM). (H–J) Evaluating MMP-2 and MMP-9 levels through Western blot (*p < 0.05, **p < 0.01, ***p < 0.001).

Next, we explored whether GA inhibits ESCC malignant progression by modulating the IL-6/STAT3/Notch pathway. Following IL-6 treatment, the cell viability of ESCC cells was notably increased, but this effect was reversed when GA was added, whereas both S31-201 and DAPT could further enhance the effect of GA (Fig. 3A). Clone formation experiments revealed that IL-6 treatment markedly increased cell clone formation rates, and the addition of GA weakened the effect of IL-6, whereas either S31-201 or DAPT further reduced the clone formation rate (Fig. 3B). Through flow cytometry, we found that GA caused a notable rise in G1 phase and S phase cell numbers, and a marked decline in the G2 phase, suggesting that GA led to cell arrest in the G1 and S phases, the addition of S31-201 or DAPT further caused cells to arrest in the G1 phase (Fig. 3C–E). The impact of GA on cell invasion and migration was evaluated by Transwell. The amount of ESCC cells that invaded and migrated to the lower chamber (purple) after IL-6 treatment was significantly increased (Fig. 3F–G). The addition of GA reduced the amount of invasive and migrating cells, and S31-201 or DAPT could further improve the suppressive impact of GA on cell invasion and migration. Not only that, Western blot revealed that MMP-2 and MMP-9 levels were notably elevated after IL-6 treatment, whereas the impact of IL-6 was weakened after adding GA, S31-201 or DAPT could further enhance the effect of GA (Fig. 3H–J). The above results indicate that high levels of IL-6 can promote the malignant progression of ESCC by triggering the STAT3/Notch pathway, while GA can inhibit the malignant biological properties of ESCC by inhibiting the IL-6/STAT3/Notch pathway.

### GA promotes ESCC cell apoptosis and oxidative stress via IL-6/STAT3/Notch pathway

**Figure 4:**
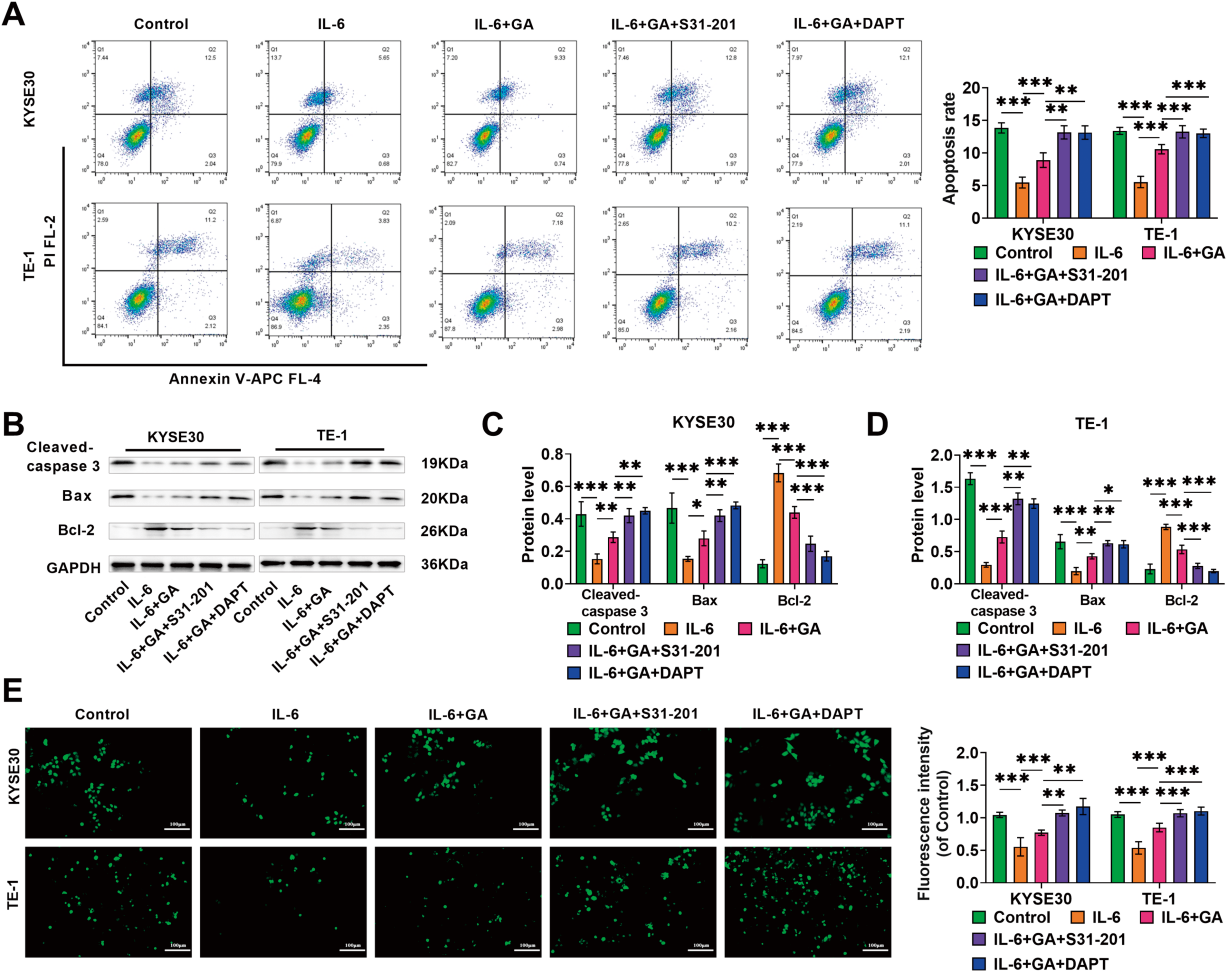
GA induces apoptosis and oxidative stress in ESCC cells via IL-6/STAT3/Notch pathway. (A) Flow cytometry examined apoptosis rates of KYSE30 and TE-1 cells. (B–D) Western blot detected Bcl-2 and apoptosis marker proteins Cleaved-caspase 3 and Bax levels of different treatment groups. (E) Using a DCFH-DA fluorescent probe, ROS levels in ESCC cells were assessed (20×, bar = 100 μM) (*p < 0.05, **p < 0.01, ***p < 0.001).

After IL-6 treatment, the apoptosis rate of KYSE30 and TE-1 cells was markedly reduced, while the addition of GA notably elevated the apoptosis rate, the addition of S31-201 or DAPT could further enhance the effect of GA (Fig. 4A). Not only that, IL-6 treatment caused a notable decline in apoptosis-linked proteins Cleaved-caspase 3 and Bax levels and a marked rise in anti-apoptotic protein Bcl-2 level, and GA weakened the impact of IL-6, while the addition of S31-201 or DAPT could further promote cell apoptosis, aligning with the results from the flow cytometry analysis (Fig. 4B–D). Additionally, the impact of GA on oxidative stress in ESCC cells was examined using a DCFH-DA fluorescent probe. IL-6 caused a notable decrease in ROS levels in ESCC cells. The effect of IL-6 was weakened after adding GA while adding S31-201 or DAPT could further increase ROS levels (Fig. 4E). The above results suggested that high levels of IL-6 hindered the apoptosis and oxidative stress of ESCC cells by triggering the STAT3/Notch pathway, whereas GA promotes cell apoptosis and oxidative stress by inhibiting the IL-6/STAT3/Notch pathway.

### GA enhances chemosensitivity of ESCC cells to DDP via IL-6/STAT3/Notch pathway

**Figure 5:**
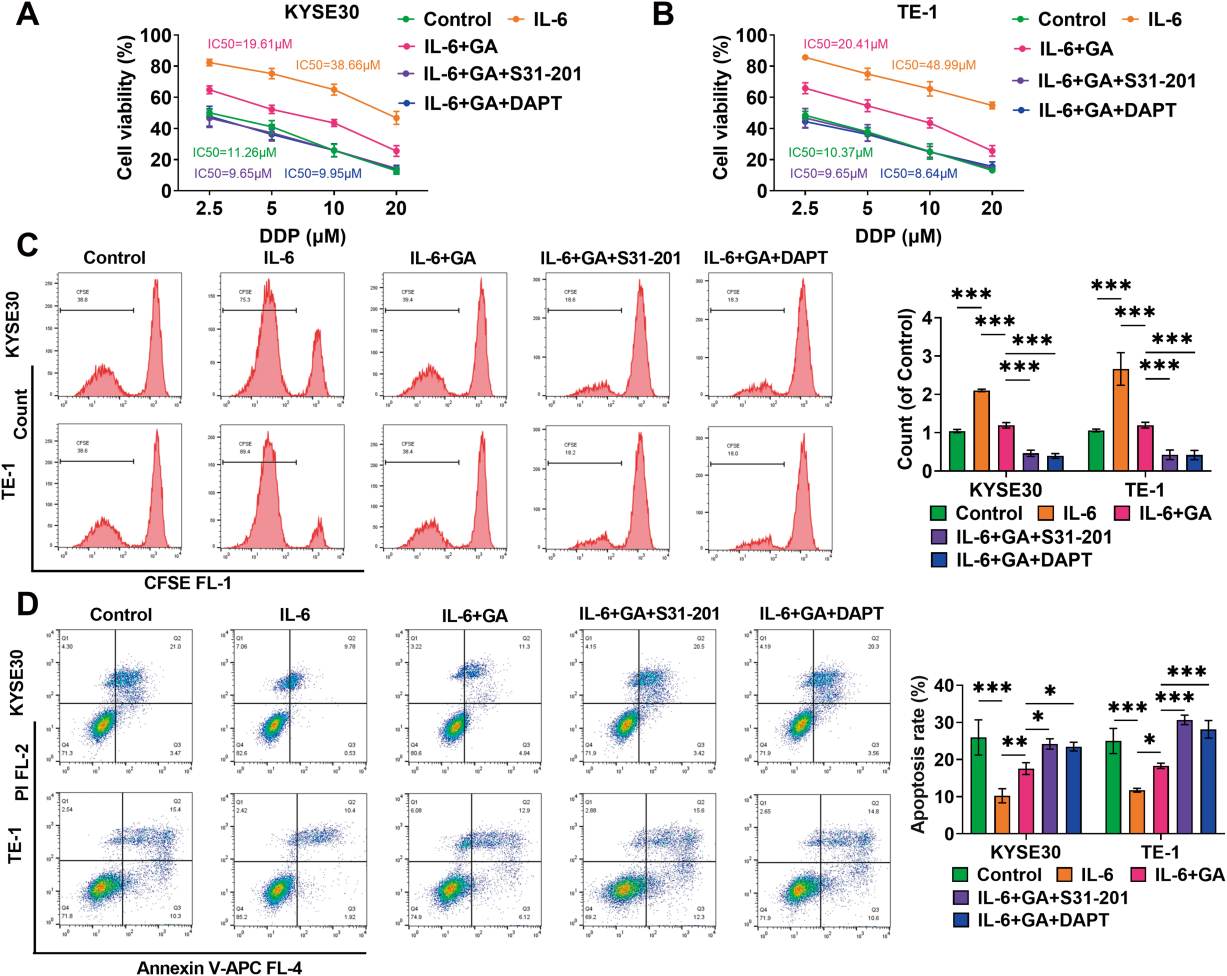
GA enhances the chemosensitivity of ESCC cells to DDP via IL-6/STAT3/Notch pathway. (A and B) CCK-8 assay detected the IC50 values of TE-1 and KYSE30 cells against DDP. (C) Cell proliferation was assessed by a CFSE fluorescent probe. (D) Flow cytometry measured apoptosis rates after different treatments (*p < 0.05, **p < 0.01, ***p < 0.001).

The IC50 values of ESCC cells against DDP were examined through CCK-8 assay, and the proliferation rate of the cells in each group decreased significantly with the increase of DDP concentration, indicating that DDP could effectively inhibit cell proliferation. The IC50 of KYSE30 and TE-1 cells were 11.26 and 10.37 μM, respectively, and were elevated to 38.66 and 48.99 μM after IL-6 treatment, whereas GA was able to reduce the IC50 of the cells, which was further reduced by the addition of either S31-201 or DAPT (Fig. 5A and B). Based on the IC50 values of KYSE30 and TE-1 cells against DDP, we chose a concentration of 10 μM DDP for subsequent experiments. Next, CFSE fluorescence staining showed that IL-6 increased cell proliferation after DDP treatment, while GA partially attenuated proliferative ability, and the addition of either S31-201 or DAPT further inhibited cell proliferation (Fig. 5C). Not only that, we found that IL-6 treatment notably declined the apoptosis rate of KYSE30 and TE-1 cells after DDP treatment, adding GA partially increased the apoptosis rate and adding S31-201 or DAPT could further improve the apoptosis rate (Fig. 5D). These results showed that IL-6 improved DDP resistance, resistance was reduced and the apoptosis rate was increased after the addition of GA, while S31-201 or DAPT could further reduce the cell resistance to DDP, indicating that GA could increase the DDP sensitivity of ESCC cells through IL-6/STAT3/Notch pathway.

### GA enhances DDP chemosensitivity in ESCC graft tumors

**Figure 6:**
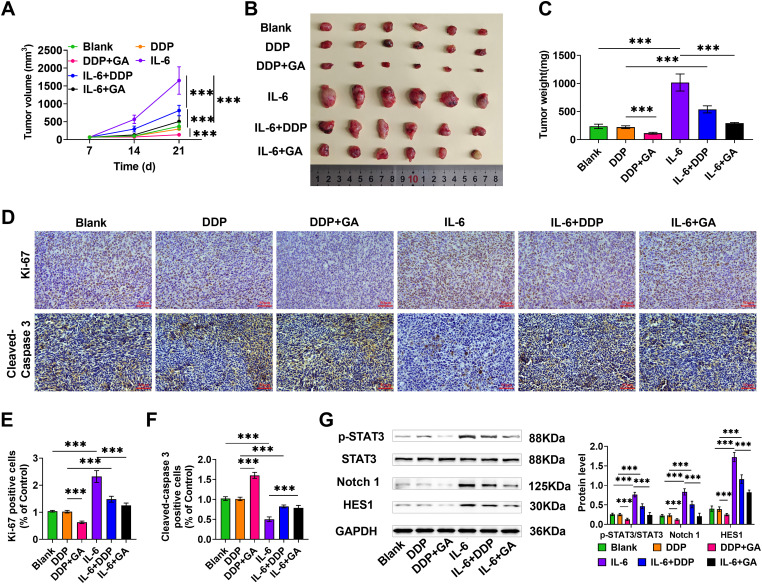
GA enhances DDP chemosensitivity in ESCC graft tumors. Mice were divided into model groups (Blank), DDP group, DDP+GA group, IL-6 group, IL-6+DDP group, and IL-6+GA group (n = 6) in a random manner. The drug was administered once every 3 days. The size of tumors was assessed on days 7, 14, and 21 with vernier calipers, and tumor volume (A) was calculated. After 21 day of drug administration, mice were anesthetized and euthanized, tumors were excised and photographed (B), and tumor weights were measured (C). (D–F) Immunohistochemical assessed Ki-67 and Cleaved-caspase 3 expression (40×, bar = 50 μM). (G) Examining p-STAT3/STAT3, Notch 1, and HES1 levels by Western blot (***p < 0.001).

Finally, to explore the impact of GA on the sensitivity of DDP in tumors *in vivo*, we constructed TE-1/DDP-resistant cells and subcutaneous graft tumor models in mice. Following the injection of IL-6, there was a notable increase in both tumor volume and weight and the injection of either IL-6+GA or IL-6+DDP partially reduced tumor volume and weight. Importantly, the combination of DDP and GA injections had a much greater suppression of tumor growth compared to using DDP alone, indicating that GA boosts the DDP sensitivity of TE-1/DDP cells (Fig. 6A–C). Immunohistochemical findings demonstrated that the levels of Ki67 after DDP+GA injection were significantly lower than that of DDP alone, whereas Cleaved-caspase 3 level was inversely related to those of Ki-67, suggesting that GA enhanced the inhibitory impact of DDP on tumor cell proliferation. After IL-6 injection, Ki-67 expression was significantly elevated and Cleaved-caspase 3 was significantly decreased, which was partially ameliorated by either GA or DDP injections (Fig. 6D–F). The level of STAT3 phosphorylation as well as Notch1 and HES1 proteins were significantly reduced in ESCC tissues after DDP+GA injection compared to DDP alone. Not only that, injection of IL-6 activated the STAT3/Notch pathway, and either GA or DDP partially reduced the effect of IL-6 (Fig. 6G). These results further indicated that GA enhanced the DDP sensitivity of TE-1/DDP cells *in vivo* and attenuated the promotion of IL-6 on tumor growth, implying that it enhanced the DDP sensitivity of TE-1/DDP cells through IL-6/STAT3/Notch pathway.

## Discussion

ESCC, with its insidious onset and lack of precision treatment, has been the primary reason for cancer deaths worldwide [29,30]. DDP chemotherapy is an optimal choice for most patients with end-stage ESCC; however, continuous chemotherapy can easily lead to drug resistance in ESCC cells, severely limiting the treatment outcome. Therefore, it is urgent to find therapeutic strategies that can improve DDP sensitivity.

GA has multiple biological activities and has broad application prospects in anti-cancer, improving diabetic nephropathy, and preventing osteoporosis [31–33]. It has multiple targets and its function in inhibiting cancer malignant advancement has attracted much attention. Hong et al. discovered that GA hindered the capacity of MCF7 breast cancer cells to migrate and invade in acidic conditions, suppressed epithelial-mesenchymal transition, and induced apoptosis [34]. Additionally, GA suppressed the proliferation and migration of human lung cancer cells and could enhance the antitumor properties of DDP via regulating the Janus kinase/STAT3 pathway and related apoptotic molecules [35]. In addition, GA has a high level of security. Findings of *in vitro* and *in vivo* experiments have indicated no significant cytotoxicity of GA in the range of 0–100 μM [36]. Research has indicated that GA at 50, 100, and 200 μM causes DNA damage in human prostate cancer cells, inhibits DNA repair gene expression, and causes apoptosis in tumor cells [37]. In this research, GA (0–500 μM) did not exhibit a toxic effect on HEEC but could decline the viability of ESCC cells in a concentration- and time-dependent manner, leading to a decline in cell proliferation and a rise in cell apoptosis. Additionally, GA decreased the IC50 value of ESCC cells to DDP and enhanced the effect of DDP in inhibiting proliferation and inducing apoptosis, indicating that it enhanced the DDP sensitivity of ESCC, consistent with prior studies.

Chronic inflammation is a major hallmark of cancer, IL-6 acts as an important pro-inflammatory cytokine [38]. Studies have revealed a high level of IL-6 expression in the tumors and blood of ESCC patients and induce the infiltration of CD39 natural killer cells, which is connected to an unfavorable prognosis for those individuals [39]. Notably, IL-6 can activate STAT3, leading to enhanced resistance to DDP treatment in ESCC cells [40]. Song et al. showed that IL-6 treatment can trigger the Notch pathway in glandular epithelial cells, thereby promoting the progression of endometriosis [41]. Our findings demonstrated that IL-6 could activate the STAT3/Notch pathway, aligning with findings from earlier research. MMP-2 and MMP-9 participate in breaking down the extracellular matrix and are crucial for cell migration and invasion [42]. In this research, IL-6 increased MMP-2 and MMP-9 levels, promoted the malignant progression of ESCC cells, and enhanced DDP resistance. Conversely, GA attenuated the impact of IL-6, and co-treatment of GA with STAT3/Notch inhibitors further reduced the influence of IL-6, implying that GA inhibited the malignant activities of ESCC and enhanced the sensitivity of DDP chemotherapy by blocking the IL-6/STAT3/Notch pathway.

During normal metabolic processes, the body continuously produces ROS, which are consumed by antioxidant enzymes and small molecules with reducing effects, thus maintaining the dynamic balance between oxidation and antioxidation in the body [43]. Different concentrations of ROS have different effects on cells: high concentrations of ROS disrupt the integrity of the plasma membrane, cause DNA damage, and lead to impaired normal cellular signaling, while low levels of ROS may promote cell proliferation and differentiation and may induce tumorigenesis [44]. It has been pointed out that tumor cells are in a higher redox state than normal cells, making them more prone to ROS-induced damage, which leads to a certain selective killing ability of ROS on tumor cells [45]. According to reports, GA can accelerate the production of ROS in the body, which may be an important mechanism for its anti-tumor effect [46,47]. We found that high levels of IL-6 could reduce ROS levels and inhibit oxidative stress in ESCC cells. GA can increase ROS levels by hindering the IL-6/STAT3/Notch pathway, which in turn promotes ESCC cell apoptosis. Importantly, DDP can also cause the generation of excessive amounts of ROS in the body, and this commonality may be an important reason why GA is able to enhance the sensitivity of DDP chemotherapy, which deserves to be further explored in the future.

## Conclusion

GA reduces the viability and proliferative capacity of ESCC cells and promotes their apoptosis and oxidative stress. Notably, IL-6 treatment activated the STAT3/Notch pathway and increased cell resistance to DDP, whereas GA interfered with the impact of IL-6 treatment, confirming that GA enhanced the DDP sensitivity of ESCC cells by inhibiting IL-6/STAT3/Notch pathway. This study provides a theoretical basis to clarify how GA improves the responsiveness of ESCC cells to chemotherapy utilizing cellular experiments and transplantation tumor models in nude mice. However, the use of GA as a powerful chemosensitizer and its application in the clinic requires extensive and in-depth research to refine its specific mechanism of action. Additionally, subsequent studies should further explore the role of the IL-6/STAT3/Notch pathway in the drug resistance of ESCC cells and the dimerization of STAT3. Future research should delve deeper into the antioxidant and pro-oxidant effects of GA.

## Data Availability

The data supporting the findings of this study can be obtained from the corresponding author, upon request.
